# Reassessing the causal interpretation of the UK sugar-rationing natural experiment

**DOI:** 10.1073/pnas.2630921123

**Published:** 2026-10-30

**Authors:** Nicola Cirillo

**Affiliations:** ^a^ Department of Clinical and Experimental Medicine, University of Foggia, Foggia 71122, Italy

Zhu and Zhang report that early-life sugar rationing causally reduces adult cancer incidence and argue that this finding has direct implications for sugar intake during the first 1,000 d of life (1). Using the authors’ public replication materials (2) and historical records, we show that this interpretation rests on assumptions about both the timing and nature of the historical exposure that are difficult to sustain. Earlier studies (3, 4) exploiting the same natural experiment were more circumspect; for example, Zheng et al. characterized their findings as a policy-level rather than the effect of intake or exposure (4). Zhu and Zhang, however, explicitly refer to sugar intake.

First, September 1953 does not represent an abrupt transition from low to high sugar exposure (Fig. 1). Household sugar intake had already risen from approximately 41 g/d in Q1 1953 to about 63 g/d by Q3, before the September cutoff used to assign treatment, compared with approximately 72 g/d thereafter (2). This reflected a staged liberalisation: sweets derationed February 1953, the ration raised to 1 lb/wk in May, a Coronation bonus in June (5, 6). Thus, much of the exposure change preceded the identifying cutoff. Treating September 1953 as a step-function change therefore misaligns treatment assignment with the underlying exposure trajectory.

**Fig. 1.**
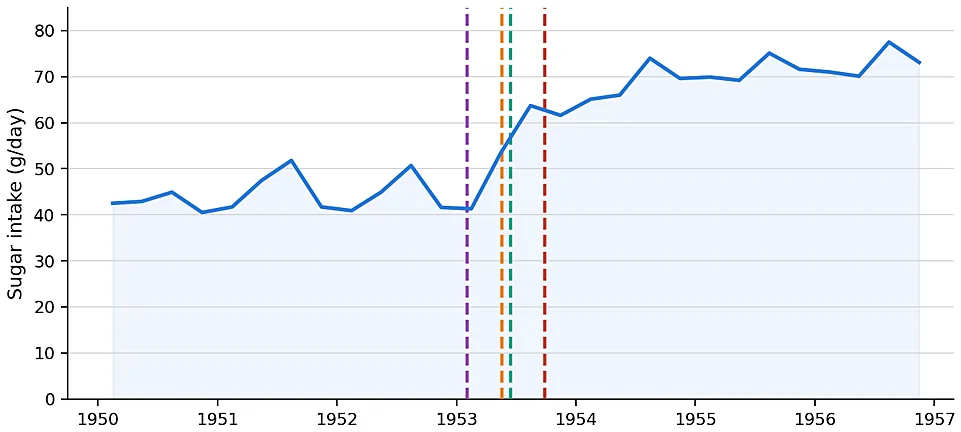
UK household sugar intake, 1950–1956 (quarterly; authors’ replication data, Dataset_1000days_Fig_S1.dta, converted at 4 kcal/g). Vertical dashed lines mark 1953 rationing relaxations: sweets derationed (purple, February), ration raised to 1 lb/wk (orange, May), Coronation bonus (green, June), and full abolition (red, 26 September)—the only event defining treatment in the study. Most of the 41 to 63 g rise precedes the red line.

Second, the assumed infant exposure is particularly problematic. Gracner et al. state that rationing implied “little to no sugar offerings” to children under two (3); Zheng et al. subsequently state that under-twos were “not allocated sugar or sweets” and that “no added sugars were permitted” (4). Zhu and Zhang similarly infer minimal infant exposure from the absence of a separate allocation (1). Yet contemporary Ministry of Food documentation contradicts the stronger interpretation. The *Welfare Foods Service*, published during the study period, states that the baby’s green ration book provided “adult rations or allowances of bacon, cheese, eggs, fats, *sugar*, *and sweets*,” albeit “initially for the mother’s benefit” (7). It also confirms provision of National Dried Milk specifically for children under two, which required 1½ teaspoons of sugar per feed (8). Therefore, the assumption about absence of access, consumption, or permission to consume added sugar by infants is dubious.

Third, the exposure was a sugar-dominated, progressive nutritional-policy change, not an isolated sugar intervention (9). Total energy increased by approximately 100 to 150 kcal/d, and nonsugar categories moved around the sugar cutoff too (Table 1: fats +12.6%, fruit/vegetables +8.8%); other foods were progressively derationed within the same birth-cohort window (2, 10). The authors themselves acknowledge that sugar-specific biological effects cannot be fully separated from higher caloric intake.

**Table 1.** Change in household consumption by food category around the September 1953 sugar cutoff [derived from authors’ public replication data (Dataset_1000days_NUTRITION_Fig_S3.dta); mean of 4 quarters before vs. 4 quarters after the cutoff]

Category	Change around Sept. 1953 sugar cutoff
Sugar (kcal/day)	+33.4%
Fats (butter + margarine + lard)	+12.6%
Fruit and vegetables	+8.8%
Dairy	+0.2%
Cereals	−0.6%
Meat (total)	−4.1%

Finally, “dose dependency” is inferred from categorical exposure-duration estimates rather than demonstrated by a formal monotonic-trend test. Some cancer trajectories are visibly nonmonotonic, particularly with small event counts. A linear-contrast or order-restricted test, together with placebo and alternative cutoffs around the earlier 1953 relaxations, would directly test the proposed dose–response.

None of this overturns the natural experiment as an estimate of the rationing regime’s long-term association. But the central claim about causal effects of a reduction in sugar intake or exposure during specific developmental windows remains unsupported.

## Data Availability

There are no data underlying this work.
